# Native Amazonian *Canga* Grasses Show Distinct Nitrogen Growth Responses in Iron Mining Substrates

**DOI:** 10.3390/plants10050849

**Published:** 2021-04-22

**Authors:** Cecilio F. Caldeira, Madson O. Lima, Silvio J. Ramos, Markus Gastauer

**Affiliations:** Instituto Tecnológico Vale, Rua Boaventura da Silva 955, Belém CEP 66055-090, Pará, Brazil; madson.olima@gmail.com (M.O.L.); silvio.ramos@itv.org (S.J.R.); markus.gastauer@itv.org (M.G.)

**Keywords:** *Paspalum cinerascens*, *Axonopus longispicus*, Poaceae, rock outcrops, land degradation, rehabilitation

## Abstract

Native species may have adaptive traits that are advantageous for overcoming the adverse environmental conditions faced during the early stages of mine land rehabilitation. Here, we examined the nitrogen (N) growth responses of two native perennial grasses (*Axonopus longispicus* and *Paspalum cinerascens*) from *canga* in nutrient-poor iron mining substrates. We carried out vegetative propagation and recovered substantial healthy tillers from field-collected tussocks of both species. These tillers were cultivated in mining substrates at increasing N levels. The tillering rates of both species increased with the N application. Nonetheless, only in *P. cinerascens* did the N application result in significant biomass increase. Such growth gain was a result of changes in leaf pigment, stomatal morphology, gas exchanges, and nutrients absorption that occurred mainly under the low N additions. Reaching optimum growth at 80 mg N dm^−3^, these plants showed no differences from those in the field. Our study demonstrates that an input of N as fertilizer can differentially improve the growth of native grasses and that *P. cinerascens* plants are able to deposit high quantities of carbon and protect soil over the seasons, thus, making them promising candidates for restoring nutrient cycling, accelerating the return of other species and ecosystem services.

## 1. Introduction

Environments undergoing intensive landscape transformations and the exposure of large quantities of raw geologic materials (e.g., mining, roads, railways, and dam construction) with poor soil structure and low organic matter and nutrient availability may receive support in the form of technical measures to improve the rehabilitation processes [1,2]. Under such conditions, active rehabilitation measures, i.e., planting and seeding of diverse species mixtures, intend to accelerate the return of key ecosystem functions (soil protection, nutrient cycling, and water supply) [3]. Fast-growing species (frequently a seed mixture of agricultural grasses and legumes) have been used as part of the conventional rehabilitation techniques that are commonly applied to build up ground cover and biomass incorporation, which are fundamental steps during the beginning of a rehabilitation process when environmental conditions may hamper plant growth [1,4,5]. Nonetheless, such rehabilitation measures would be more advantageous by applying native species, which are expected to reduce the risk of biological invasions, have adaptations to local environmental conditions, contribute to interaction networks (pollinators and dispersers), and play important roles in community stability and ecosystem function restoration [6,7,8], which have become urgent necessities in the current climatic scenario [9].

On the plateaus of Serra dos Carajás (eastern Amazon), one of the largest high-grade iron ore reserves in the world [10], surface mining is accompanied by the challenge of rehabilitating large areas that have undergone severe degradation. Moreover, despite the benefits of replanting and the legal requirements related to offsetting biodiversity loss [11], very little information can be found in regard to the selection, propagation, and establishment of distinct plant communities of *canga* physiognomies [12,13,14], a rich savanna-like vegetation type growing over iron-rich substrates at the tops of mountains [15,16]. This open vegetation type can face a series of adverse environmental conditions, in particular, high UV radiation exposure, elevated daily temperatures, nutrient depletion in poorly developed soils, low water retention capacities, and prominent drought periods [10], and therefore possibly carries adaptive traits that could be conducive to successful mine land rehabilitation.

In a recent inventory of all plateaus of Serra dos Carajás, Poaceae was identified as the richest among the 116 registered angiosperm families, with 86 (out of 856) species [17]. Overall, grasses are recognized for their adaptations to warm and dry environments, including high carbon assimilation and water use efficiency [18,19]. The two grass species *Axonopus longispicus* and *Paspalum cinerascens* were the most common and were found to be widely distributed, covering large open areas of grasslands and identified amid the *canga* shrublands [20]. Both species are perennial, have fast growth rates during the wet season, form large bunches, and are not restricted to *canga* physiognomies [20]. These native grasses could be used to build strips over steep slopes inside mines and on piles of waste substrates. Grass strips can be effective for soil conservation and rehabilitation by reducing runoff, capturing eroded materials, and, together with biomass deposition, reducing nutrient loss and enhancing fertility [21]. Moreover, the expected high iron tolerances of both species, which have already been reported for both genera [22,23], are of special advantage, as the high iron availability remaining in mining waste substrates is a significant challenge to rehabilitation processes, in addition to the low natural fertility of these substrates [13,24].

The general low fertility of iron ore mine land substrates requires the input of nutrients to improve plant growth [13,25]. Nitrogen (N) is the nutrient that plants require in the highest quantity after carbon; nitrogen can reach 1–5% of total plant dry matter [26]. Nitrogen supplies are essential to ensure the growth and productivity of grass species, especially in environments that have been depleted of this element [26,27]. Nitrogen is involved in a myriad of cellular processes (building proteins, nucleic acids, chlorophyll, etc.), is a determinant in overcoming tiller bud dormancy (by affecting endogenous hormone regulation [28]), and promotes and ensures plant growth and whole-plant biomass and yield [27,28,29]. Nitrogen fertilization is also the major variable cost after switchgrass establishment, and the correct N supply necessary to maximize biomass yield (the optimized N response) can define net returns and prevent environmental problems [29,30]. In fact, N fertilizer overuse imposes a series of environmental disruptions, from eutrophication (leaching to groundwater) to air pollution (increment of global warming gases), biodiversity loss [31,32], decreased N use efficiency, and increased economic costs [26,27,32]. As crop species are able to absorb approximately 40–50% of the applied N [33], and there is large variability in optimum N supplies even among different genotypes and environmental conditions [27,34], well-balanced N fertilization is paramount for the successful integration of native grass species within mine land rehabilitation projects.

Here, we examined the N growth response of two native grass species from Amazonian *canga* (*A. longispicus* and *P. cinerascens*) in mineland substrates. We hypothesized that both species will benefit from the N input in the nutrient poor minelands from Carajás. In order to determine the most efficient nitrogen level for the establishment and maintenance of the selected grass species, tillers were grown in mining waste substrates at four N levels. Experiments were carried out simultaneously in greenhouse conditions to evaluate the following: (1) tillering, biomass production, and partitioning; (2) pigment content and gas exchanges (compared with plants from natural *canga* ecosystems); (3) stomatal distribution and size; and (4) nutrient partitioning. When applicable, regression functions were applied to growth and physiological variables to determine the optimal N dose for each species.

## 2. Materials and Methods

### 2.1. Plant Material and Propagation

Tussocks of the two perennial grass species (*P. cinerascens* and *A. longispicus*) selected for this study were collected in the *canga* plateau N1 of Serra dos Carajás because of the abundances of these species in this area [20]. Under lab conditions and after the removal of debris, the whole plants were broken apart to separate the tillers, which were cut with scissors to 10 cm lengths, and dried leaf parts were removed. Each tiller was fixed into commercial organic substrate pellets (Jiffy-7^®^) in 50 mL plastic seedling trays, irrigated with distilled water, and transferred to a plant growth chamber (Fitotron^®^ SGC 120, Weiss Technik, Loughborough, UK) with a photoperiod of 12:12 h, a photosynthetic photon flux density (PPFD) of 50 μmol m^−2^ s^−1^, a day/night temperature regime of 25/20 °C, and a constant relative air humidity of 75%. Water availability in the substrate was maintained by daily irrigation. After 30 days, the survival rates of 200 tillers (eight repetitions with 25 tillers each) of each species were examined.

### 2.2. Plant Growth Conditions

#### 2.2.1. Substrate Analysis and Preparation

The mining waste substrate was collected from a representative location of the S11D Eliezer Batista Complex, Canaã dos Carajás, Pará, Brazil. After collection, the substrate was air dried and sieved to remove particles larger than 1 cm. The physical and chemical properties of the substrate were determined after homogenization and sieving through a 4 mm mesh (Table S1). The pH was determined by a pH electrode in a 1:2.5 soil-to-water ratio, and the organic carbon was determined using the potassium dichromate (K_2_Cr_2_O_7_) method. The available P, K B, Zn, Fe, Mn, and Cu contents were determined using the Mehlich-1 method (0.05 mol L^−1^ HCl + 0.0125 mol L^−1^ H_2_SO_4_), where P was determined by colorimetry, K was determined by flame photometry, and the other elements were determined by inductively coupled plasma-atomic emission spectrometry (ICP-AES). Exchangeable Ca, Mg, and Al were measured using atomic absorption spectrophotometry on 1 M KCl extracts with the addition of lanthanum oxide [35]. The soil texture was determined, as described by [36]. Thirty days before planting, liming was carried out to raise the base saturation from 10% to 50% using commercial calcined lime (characterized by the manufacturer as having 24.9% Ca and 8.4% Mg). Additionally, a base fertilizer was applied to the substrate that consisted of 200 mg P dm^−3^ (Na_2_PO_4_H_2_O), 100 mg K dm^−3^ (KCl), 60 mg S dm^−3^, 0.5 mg B dm^−3^ (H_3_Bo_3_), 5 mg Zn dm^−3^ (ZnSO_4_ 7.H_2_O), 1.5 mg Cu dm^−3^ (CuSO_4_.7H_2_O), and 0.15 mg Mo dm^−3^ ((NH_4_)_6_ Mo7O_24_.4.H_2_O). Liming and fertilization followed the revegetation protocols from Carajás mines. To evaluate the nitrogen growth responses of *P. cinerascens* and *A. longispicus*, tillers obtained from the previous assay were cultivated in pots with volumes of 2.3 dm^3^ filled with mining waste substrate. Considering the overall recommendation for tropical grass management [37] equivalent to 80 mg N dm^−3^ and the large variability of N requirement in grasses [27,34], we applied four N levels (0, 40, 80, and 200 mg N dm^−3^, with urea (CH_4_N_2_O) as the source) aiming to determine the optimum N supply to both species. Each treatment was composed of eight repetitions, and one pot received a single tiller. Nitrogen fertilization was carried out at regular intervals of 30 days (0, 30 and 60 days) by dividing each dose into three applications.

#### 2.2.2. Environmental Conditions

The plants were cultivated under greenhouse conditions for 110 days beginning when they were transferred from the seedling trays to the pots with the mining waste substrate. The temperature and relative air humidity were monitored every 15 min with a thermocouple connected to a datalogger (RHT10, Extech Instruments, Boston, MO, USA). During this period, the daily air temperature varied between 25 and 37 °C, and the vapor pressure deficit varied between 0.4 and 2.5 kPa (Figure S1). The midday photosynthetic photon flux density (PPFD) was periodically measured (LI-190R, LICOR, Lincoln, NE, USA) and ranged from 1500 to 2500 μmol m^−2^ s^−1^. The water availability was kept at 70% of the soil water retention capacity by replacing water loss from evapotranspiration with distilled water after daily monitoring of the pot weights.

### 2.3. Plant Measurements

#### 2.3.1. Tillering Rate, Biomass, and Nutrient Partitioning 

The tillering rate was determined as the number of tillers produced by the plants after 110 days of cultivation. Then, the shoot and root biomasses were harvested separately, washed and oven-dried at 62 °C to a constant weight. For the macro- and micronutrient analyses, subsamples of 500 mg of dried shoot and root tissues (of 500 mg each) from each species were digested with 4.0 mL of 60% HNO3 and 2.0 mL of 70% HClO4 (Sigma-Aldrich, St. Louis, MO, USA). The total Ca, Mg, K, B, Cu, and Zn contents were determined using an atomic absorption spectrophotometer. The total S content was determined using the turbidimetry of barium sulfate, the total P was determined by using a spectrophotometer to measure the colorimetry of a phospho-molybdenum complex at 680 ηm, and the total N content was determined by the Kjeldahl method after sulfuric acid digestion.

#### 2.3.2. Gas Exchanges

The gas exchanges were measured in the fully developed leaves of the main stem of each plant with a gas analyzer (LI-6400XTR, LICOR, Lincoln, NE, USA) two days before plant harvesting. The measurements were carried out in the morning (between 9:00 and 11:30, local time UTC 3:00), and the environmental conditions inside the cuvette were adjusted to a PPFD of 1000 μmol m^−2^ s^−1^, temperature of 28 °C, a vapor pressure deficit of 1.8 kPa, and an air CO_2_ concentration of 400 ppm. The water use efficiency (WUE) was calculated from the gas exchanges measurements as the ratio between the carbon assimilation rate (A, μmol CO_2_ m^−2^ s^−1^) and transpiration rate (E, mmol H2O m^−2^ s^−1^) once the vapor pressure deficit was held constant during the measurements. The gas exchanges were also measured under field conditions from 10 plants of each species growing on the *canga* plateau N1 of Serra dos Carajás. These measurements were also carried out in the morning during the wet/growing season.

#### 2.3.3. Pigment Contents

The chlorophyll (a and b) and carotenoid contents were determined spectrophotometrically from leaf samples by preparing acetone extracts, as described by [38]. The leaf samples were fast-frozen in liquid nitrogen, and then stored at −80 °C. The extracts were prepared with 10 mL of 80% acetone (Sigma-Aldrich), and 1 g of each leaf sample was macerated in nitrogen liquid with a mortar and pestle. The extracts were centrifuged at 3000× *g* rpm (Eppendorf centrifuge 5810 R, Rotor A-4-81, Hamburg, Germany) for 20 min at 4 °C. The upper green solution was collected, and the absorbances of the samples were determined using a spectrophotometer (Biochrom Libra Model S80, Cambridge, UK) at the wavelengths of 470, 647, and 663 ηm. Each sample was taken as a technical triplicate, and the results were calculated following the equations proposed by Wellburn (1994).

#### 2.3.4. Stomatal Frequency and Size

The stomatal frequency, length, and width were measured on samples collected from the middle parts of the adaxial and abaxial surfaces by the impression method using nail varnish. Five impressions were taken from both sides of fully expanded leaves of each plant. Four microscope fields of 0.23 mm^2^ per leaf were counted, thus, 20 fields were counted on each leaf surface of each plant. To determine the stomatal frequency, the number of stomata per field of view was converted to the number of stomata mm^−2^. Five measurements of stomatal length and width were taken from each field, totaling one hundred stomata measured per plant. All measurements were made randomly over the slide. Micrographs and measurements were obtained with a Zeiss Axio Imager M2 light microscope coupled with an AxioCam MRC 5 camera by using Axiovision SE64 software.

### 2.4. Data Analysis

All data analyses were conducted in R version 3.5.2 [39]. Differences in the tillering survival rates between the two species were tested using one-way analysis of variance (ANOVA) followed by subsequent Student’s *t*-test. Calibration curves of all variables versus nitrogen levels were generated using a simple linear regression analysis (glm() function) in the R environment. When applicable, the optimum N level was estimated after the model derivation was completed. The figures were built using the ggplot package.

## 3. Results

### 3.1. Plant Propagation and Growth

**Figure 1:**
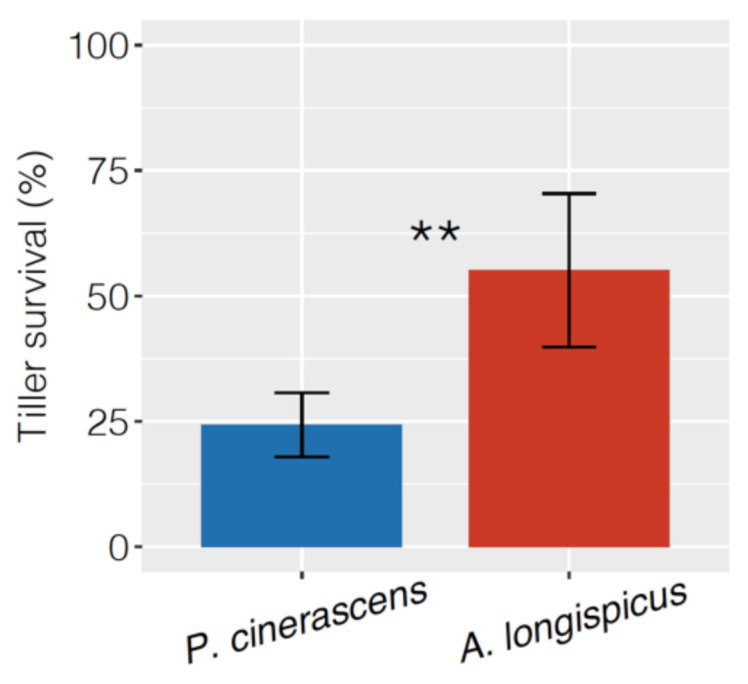
Vegetative propagation of *Paspalum cinerascens* and *Axonopus longispicus*. Percentage of tiller survival after 30 days of growth in controlled conditions (see Materials and Methods). The error bars represent the means and confidence intervals at *p* < 0.05 (*n* = 8 plots with 25 tillers each). ** represents significant differences after Student’s *t*-test at *p* < 0.01.

**Figure 2:**
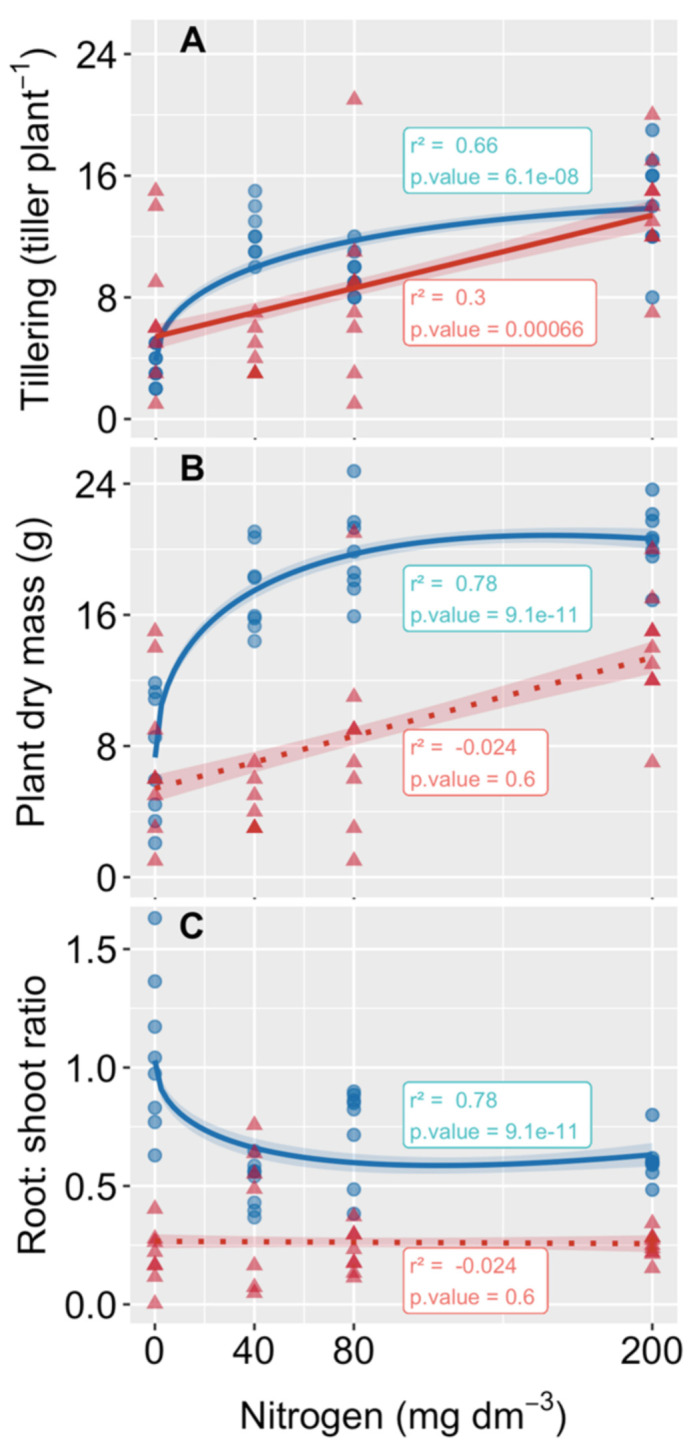
Nitrogen growth response of two native grass species from *canga* in Serra dos Carajás (eastern Amazon) grown in mining waste substrate. The tillering rate, plant dry mass, and root/shoot ratio of *Paspalum cinerascens* (circles, blue) and *Axonopus longispicus* (triangles, red) are shown. The lines correspond to the best fitted model, and the shaded areas correspond to the confidence intervals. The solid and dotted lines indicate significant and non-significant models, respectively. The r-squared value and *p*-value are shown in the boxes close to each model and the other regression parameters are shown in the Tables S2 and S3.

Tillers of both species were successfully obtained through vegetative propagation. After 30 days in controlled conditions, 55% of the *A. longispicus* tillers remained alive, more than twice the 25% survival rate obtained for the *P. cinerascens* tillers (Figure 1). After transferring the tillers to the mining waste substrate, the tillers of *P. cinerascens* showed substantial growth gains with N supplies, while this benefit was not observed for the *A. longispicus* tillers (Figure 2). In plants of *P. cinerascens*, the addition of N led to higher tillering rates (Figure 2A) that were accompanied by increased total biomasses (Figure 2B). These increments were markedly higher at the lower N level (40 mg N dm^−3^) and reached an optimum gain in the substrate close to 80 mg N dm^−3^. In fact, at this N level, the total biomass accumulated by *P. cinerascens* was three times higher than that in the control treatment. The tillering rate described a pattern similar to that of the biomass, except for a slight tillering increase sustained by nitrogen application (Figure 2A). Consequently, most of this biomass gain was translated into shoot growth, which can be observed as a significant reduction in the root/shoot ratio (Figure 2C). In contrast, plants of *A. longispicus* did not show biomass gain (Figure 2B) or alterations in the root/shoot ratio with the N supply (Figure 2C), despite a linear increment of the tillering rate, which reached twice the tiller number of the control plants in the substrate with 200 mg N dm^−3^ (Figure 2A). Additionally, the root biomass accumulated by *A. longispicus* plants was very small and led to low root/shoot ratios in all treatments. These root values were inferior to those of *P. cinerascens* in all corresponding treatments (Figure S2).

### 3.2. Gas Exchanges and Pigment Contents

**Figure 3:**
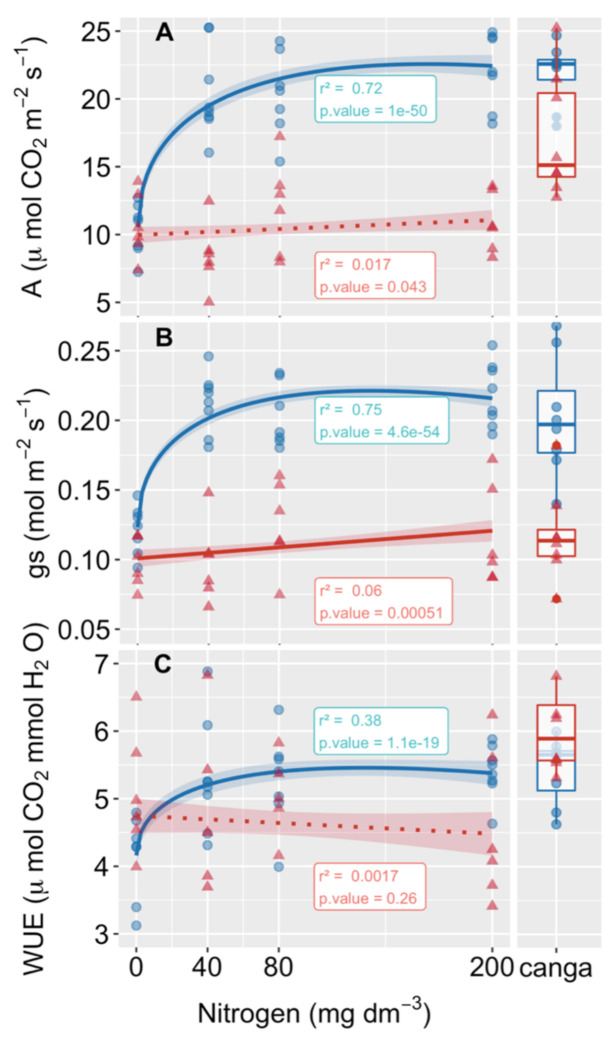
Effects of nitrogen increases in the mining waste substrates on the leaf gas exchanges of two native grass species from *canga* in Serra dos Carajás (eastern Amazon). The carbon assimilation rates (**A**), stomatal conductance (**B**), and water use efficiencies (**C**) of *Paspalum cinerascens* (blue) and *Axonopus longispicus* (red) are shown. The lines correspond to the best fitted model, and the shaded areas correspond to the confidence intervals. The solid and dotted lines indicate significant and non-significant models, respectively. The r-squared value and *p*-value are shown in the boxes close to each model and the other regression parameters are shown in the Tables S2 and S3. The boxplots correspond to measurements carried out on field-growing plants on the *canga* plateau N1 of Serra dos Carajás.

The carbon assimilation values of *P. cinerascens* and *A. longispicus* showed no significant differences when the plants were grown without N fertilization (control treatment, Figure 3A and Figure S3). With an increasing N supply, the carbon assimilation of *P. cinerascens* increased promptly and, as also observed for the total biomass, reached an optimum value in the substrate close to 80 mg of N dm^−3^ (Figure 3A and Table S2). For *P. cinerascens*, the values of carbon assimilation, stomatal conductance, and water use efficiency (WUE) showed no significant differences from plants growing in their native *canga* fields during the wet season (boxplots in Figure 3). Similar to carbon assimilation, the stomatal conductance increased with N supply and reached an asymptote above 80 mg of N dm^−3^ (Figure 3B). The WUE also increased with N, with higher values than those observed in the control treatment (Figure 3C). However, in the absence of any N addition to the substrate (control treatment), plants of *P. cinerascens* reached only half of the carbon assimilation, stomatal conductance, and WUE values as compared with plants naturally growing in *canga*. For *A. longispicus*, the gas exchange results did not show significant changes with the N supply to the substrate (Figure 3). As compared to plants in the field, the carbon assimilation of the propagated plants of *A. longispicus* was inferior, the stomatal conductance showed no difference, and the WUE was reduced in all N treatments in the mining waste substrate.

The leaf pigments (chlorophyll *a* and *b* and carotenoids) displayed a consistent linear increase with the N supply, and no significant variations in the chl*a*/*b* ratio were observed for *P. cinerascens* plants (Figure S4 and Table S2). However, as observed for other variables, no significant variations in pigment contents were observed in *A. longispicus* plants in any treatment (Figure S4 and Table S3).

### 3.3. Root and Shoot Nutrient Contents

**Figure 4:**
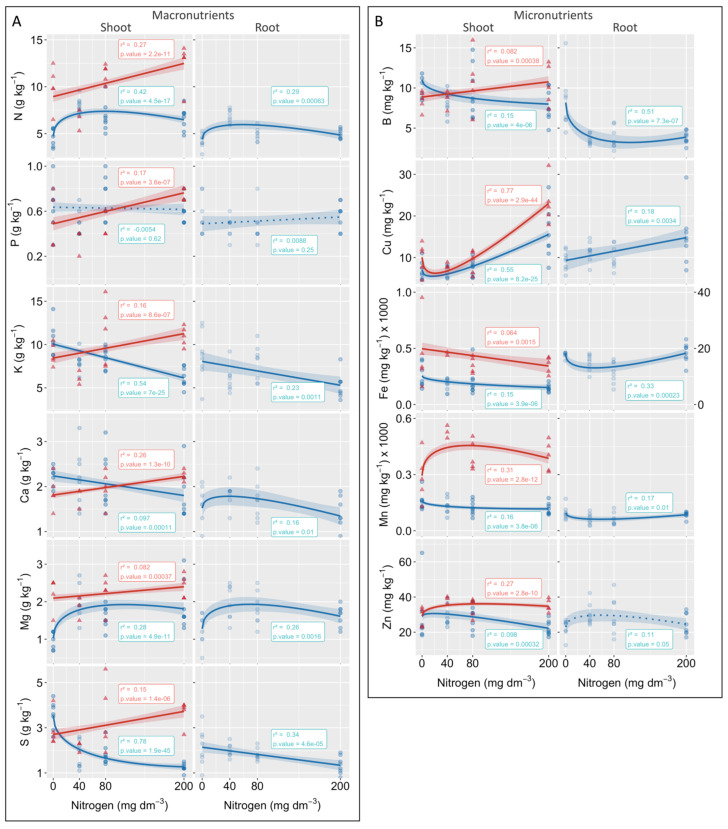
Effects of nitrogen increases in the mining waste substrates on the shoot and root nutrient contents of two native grass species from *canga* in Serra dos Carajás (eastern Amazon). The macro- (**A**) and micronutrients (**B**) of *Paspalum cinerascens* (blue) and *Axonopus longispicus* (red) are shown. The nutrient contents were not determined in the roots of *A. longispicus* because of the lower biomasses obtained in all treatments. The lines correspond to the best fitted model, and the shaded areas correspond to the confidence intervals. The solid and dotted lines indicate significant and non-significant models, respectively. The r-squared value and *p*-value are shown in the boxes close to each model and the other regression parameters are shown in Tables S2 and S3.

As observed for the biomass and gas exchanges, the N (root and shoot) and Mg (root and shoot) contents of *P. cinerascens* showed significant increases at the lower N levels and reached maximums before the higher N levels were applied to the substrates (Figure 4A). These N increments did not affect P accumulation in shoots or roots but continuously decreased K accumulation in both shoots and roots. Such a linear decrease was also observed for Ca and S in the roots, and sharp decreases in S (shoot) and B (root) at lower N levels were also seen (Figure 4B). Increases in Cu were also observed in both plant parts, and higher contents were obtained at the higher N level (Figure 4B). Although all the other nutrients showed no significant or little changes in their contents, a very high Fe quantity accumulated in the roots (16,000 mg kg^−1^) and shoots (~190 mg kg^−1^) of the plants; in fact, most of the Fe absorbed by the plants were retained in their roots in which tissues accumulated almost 100× more Fe than did the shoot tissues.

In plants of *A. longispicus*, the nutrient contents were not determined in the roots because of the lower biomass accumulated in all treatments. In the shoots, Cu was one of the few nutrients that showed a high increase with the addition of N, and this increase was particularly observed at the higher N level (Figure 4B). Mild increases were also observed in other nutrients, such as N, P, K, Ca, and S, and high Mn contents were observed for the lower N levels, reaching more than 500 mg kg^−1^ of dry biomass when 40 mg N dm^−3^ was applied to the substrate. Moreover, as observed for *P. cinerascens*, plants of *A. longispicus* also accumulated high Fe quantities in their shoot tissues (~453 mg kg^−1^).

### 3.4. Stomatal Frequency and Size

**Figure 5:**
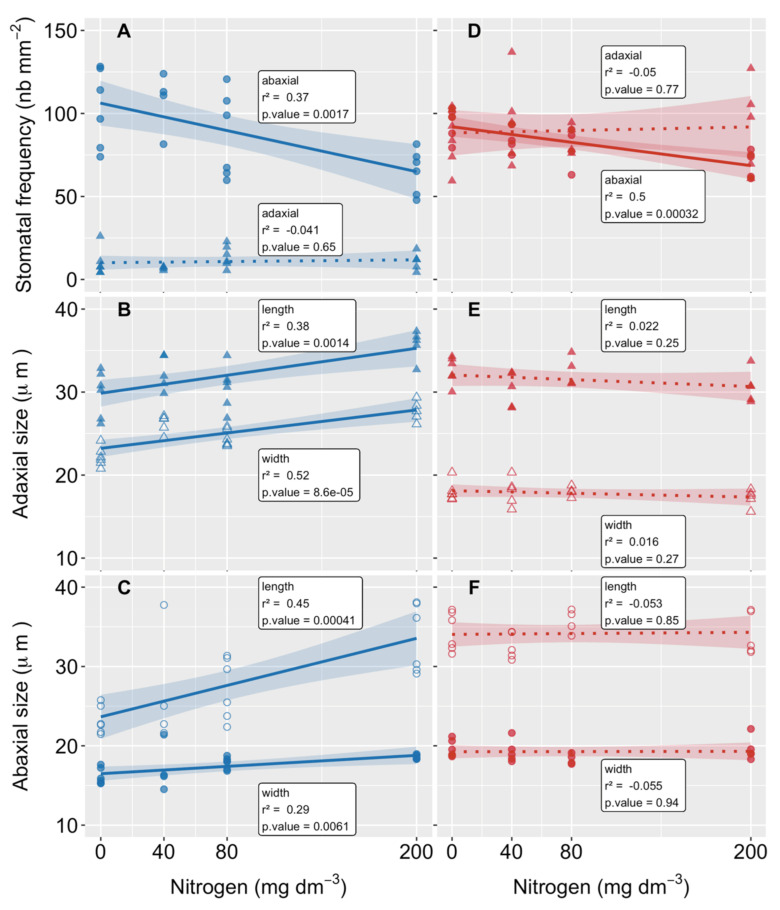
Nitrogen effects on the leaf stomatal morphologies of two native grass species from *canga* in Serra dos Carajás (eastern Amazon) grown on mining waste substrates. The stomatal frequency (**A**,**D**) and stomatal size (length and width) on the adaxial (**B**,**E**) and abaxial (**C**,**F**) leaf surfaces of *Paspalum cinerascens* (blue) and *Axonopus longispicus* (red) are shown. The lines correspond to the best fitted model, and the shaded area corresponds to the confidence intervals. The regression parameters are shown in Tables S2 and S3.

Stomata were found with different frequencies on both the upper (adaxial) and lower (abaxial) leaf surfaces (amphistomatous) in the two species. While most stomata were observed on the abaxial surface of *P. cinerascens*, an equivalent number of stomata per area was found on both leaf surfaces of *A. longispicus* (Figure 5A,D). Under the control conditions (no N addition), an average of 10 stomata mm^−2^ was found on the adaxial surface of *P. cinerascens*, 10× fewer than the 100 stomata mm^−2^ found on the abaxial surface, close to the frequency found on of both leaf surfaces of *A. longispicus*. The addition of N to the substrate led to a linear stomatal frequency decrease on the abaxial leaf surfaces of both species; the lowest values were reached at the high dose of N. No significant changes were observed on the leaf adaxial surface of either species.

In plants of *P. cinerascens*, the N supply led to an overall increase in stomatal size (length and width) on both leaf surfaces (Figure 5B,C). These variations were not significant in *A. longispicus* plants (Figure 5E,F).

## 4. Discussion

### 4.1. The Tillering Rates of Both Species Increased with N Addition, but Only P. cinerascens Plants Increased in Biomass

Nitrogen fertilization was an effective way to improve tillering in the two native grass species from eastern Amazon *canga* physiognomies. As observed in other grasses [28,29,40,41], N also stimulated bud emergence and growth from the main shoots of *P. cinerascens* and *A. longispicus*, generating new tillers that developed their own roots and were able to grow independently. This propagation strategy, combined with an N supply, can be used to accelerate tillering and propagate these native grasses in nursery conditions, as the seed availabilities and information on the physiological qualities of the plants are still missing. Despite both species being able to develop reproductive tillers and flowers and produce seeds [20], field observations also suggest that their perenniality and survival during drought events are mainly ensured by plant dormancy, a common trait seen in other perennial grasses that face adverse environmental conditions [42]. In *canga*, the seasonal severe warm and drought period requires such a strategy from several species [10]. The propagation and growth of these species resume when the rainfall season restarts and tiller buds are released from dormancy. When this happens, the reserves accumulated in the crowns and rhizomes are allocated to sustain the initial growth of the plants and the formation of new meristems [42]. As shown in this study, a substantial number of tillers collected in the field can survive bunch breakage and tiller separation under controlled conditions. In fact, we can expect more successful propagation by using plants growing in favorable environmental conditions, markedly by the correct N input for sustaining high tillering rates and biomass accumulation [30,40,42], than by using plants found in the stressed *canga* physiognomies. In accordance, approximately 20 vigorous tillers per plant were obtained from plants of *P. cinerascens* when cultivated for six months in a nutrient-rich substrate (a mixture of loam soil, organic matter, sand, and fertilizer) under greenhouse conditions (Figure S5). Furthermore, this increase in tillering rate improves surface cover and may act as an important obstacle for runoff and erosion processes [43,44,45].

Although N application is associated with tillering, biomass accumulation, and yield in perennial [30,40,46] and seasonal grasses [27,28,29,34], this relationship was only observed in plants of *P. cinerascens*. Such differences between species can be linked to the larger root investments of *P. cinerascens* than *A. longispicus*. In fact, by increasing the root biomass, *P. cinerascens* increases its ability to absorb nutrients and water, consequently, there is more material to supply the growth of the aboveground part. In these plants, even low quantities of N added to the mining waste substrate were able to improve tillering and biomass accumulation, with an optimum addition value (80 mg of N dm^−3^, equivalent to 160 kg of N ha^−1^) close to the overall recommendation for grass forage establishment and management in tropical areas [37] for switchgrass biomass production [40]; however, the observed optimum value was largely superior to the optimum of 69 kg N ha^−1^ recommended for some other grasses [30]. Actually, the optimum N level for maximizing the biomasses of grasses can be largely variable, depending on the species, soil nutrient availability, and management practices [27,34]. Furthermore, N fertilizer must be carefully managed to avoid waste, pollution, and weed population recruitment [47], especially when applied to less responsive species [46], as observed for *A. longispicus* in this study.

### 4.2. Biomass Gain Was a Consequence of Enhancing Carbon Assimilation Mediated by Leaf Pigment and Nutrient Absorption

In *P. cinerascens* plants, unlike in *A. longispicus* plants, increased N availability in the mining waste substrate translated into more N absorption by roots and allocation to competent photosynthetic organs (leaves), allowing the plants to synthetize more photosynthetic pigments and enzymes, boosting their carbon assimilation, and driving growth, measured here as tiller emissions and root and shoot biomass accumulation. Most of the N absorbed by grass species is found in leaf mesophyll cells (up to 75%), which are mainly involved in photosynthetic processes [26,48]. The leaf N also affects the size and number of chloroplasts and is found mainly as Rubisco and other thylakoid compounds (chlorophylls) responsible for light absorption and energy transfer [22,26,49]. As is consistent with other grass species [30,40,50], the patterns of N concentrations in both studied species were followed by most of the measured variables; plants that are able to capture more of the supplied N (*P. cinerascens*) have the benefits of increased carbon fixation and biomass. Moreover, in plants of *A. longispicus*, despite their overall higher shoot N concentrations and equivalent carbon assimilation rates to the *P. cinerascens* plants in the control treatment, the slight N increase in the shoots effectively stimulated tiller meristem germination but was still ineffective in triggering significant changes in leaf pigments, carbon assimilation, and biomass.

Consistent with the trends seen for N, the other nutrients directly involved in the photosynthetic process, Mg and Cu, also had increased absorption and allocation rates to the leaves of the *P. cinerascens* plants. As a central atom of chlorophyll molecules, Mg plays a key role in energy capture and participates in the carbon fixation process in Mg-dependent reactions such as PEPcarboxylase [26], while Cu, as a redox-active element, is mostly found in chloroplasts associated with plastocyanin, a component of the electron transport chain of photosystem I [51], and is involved in a myriad of other processes along the pathways of C and N metabolism and in protection against oxidative stress [26]. The absorption of these nutrients, which occurs in concurrence with other cations such as K^+^, Ca^2+^, and even NH_4_^+^ [52], may have positively affected the photosynthetic capacity of *P. cinerascens* plants despite the reduction in K and Ca concentrations observed in the shoots and roots of the plants, respectively. Nonetheless, the increased concentration of the cation Cu^2+^ observed in the shoots of *A. longispicus* with the increasing N supply was not followed by corresponding alterations in the other nutrients or by a change in carbon assimilation.

The equivalent values of carbon assimilation observed in plants of *P. cinerascens* cultivated in the mining waste substrate and plants growing in natural conditions during the wet season suggests that the plants adapt to the new environment once the substrate has received an appropriate level of nutrient corrections, including N. The carbon assimilation rate reached values close to those found for grass crop species (maize [53], sorghum [54], and wheat [27]) and *Panicum virgatum* L., a switchgrass widely cultivated for biomass energy feedstock production [55]. Species with such carbon assimilation rates are able to produce a substantial biomass and might contribute to the incorporation of large amounts of organic carbon into the ground, enhancing nutrient cycling, and soil and water qualities, which are essential for the recruitment and establishment of other plants and the return of wildlife. Moreover, native species are usually well adapted to local environmental conditions [56]. Such adaptability, already observed for both species under natural conditions [20] and now highlighted for *P. cinerascens* in mining waste substrates, are advantageous for the colonization of degraded areas such as those found after mining in the eastern Amazon.

### 4.3. Higher WUE Benefits Plants Growing in Water-Limiting Conditions, Such as Those Found in Areas Requiring Rehabilitation

Water availability is a major abiotic constraint on plant and ecosystem production worldwide. Plants have developed a series of strategies to cope with carbon acquisition from a dry atmosphere with minor water loss from wet mesophyll cells. The high WUE (calculated as the amount of carbon gained per unit of water loss) observed in both *P. cinerascens* and *A. longispicus* represents an important adaptive trait for sustaining vegetative growth and saving water without substantial costs to carbon assimilation [57]. Applied in crop selection programs to optimize agricultural freshwater consumption [58], high WUEs are of paramount importance in environments that are subject to water shortages, such as the strong drought seasonality experienced in *canga* physiognomies [10] and the degraded areas after mining. Such adaptations mean that both species are able to manage water in exchange for carbon as efficiently as crop cereals such as maize and sorghum [54], despite growing in iron-rich mining waste substrates that are depleted in organic matter. Moreover, the increase in WUE experienced by *P. cinerascens* did not occur at the expense of biomass reduction; thus, this increase may be linked to a concomitant change in leaf stomatal frequency and size, thereby, affecting photosynthesis and transpiration.

Because gas and water exchanges in leaves are essentially under stomatal control, reductions in stomatal density together with stomatal size enlargement could be the reason for the WUE gain that markedly occurred in plants of *P. cinerascens* as more N was supplied to the mining waste substrate. As previously observed [59,60], such stomatal adjustments appear to have greater effects on transpiration than on CO_2_ fixation. Although the increased size aperture contributes to slower stomatal movement and slower responses to dehydration, small changes in the linear dumbbell-shaped stomata of grass plants can track large variations in stomatal aperture [61]. In fact, small turgor changes in the guard and subsidiary cells of dumbbell stomata lead to faster and greater opening/closing movements than make changes to the kidney-shaped stomata of nongrass species, hence increasing carbon assimilation and water use efficiency [62].

Notwithstanding the WUE gain of the *P. cinerascens* plants, the demand for root water uptake and transport to the aerial parts of the plants were also increased because of the higher stomatal conductance and the larger transpiring surfaces of the shoots, which together increased the overall water demand of the plants. Once most of the assimilated carbon had been translated into shoot biomass rather than into root biomass, revealed by the reduction in the root/shoot ratio, we expect that adjustments in the root system were able to account for the water uptake demand because roots can acclimate to changing shoot demands and heterogeneous soil water availability [63]. Despite not being directly analyzed in this study, such events have been commonly reported in grass and nongrass species; changes in root system architecture (primary or lateral root production and elongation) and root hydraulic conductivity by modulating aquaporins (a family protein involved in transmembrane water and ion transport) may adjust to optimize the water status of plants [53,64,65]. Furthermore, it has been suggested that such fine control of root hydraulics may improve plant growth performance under stress and nonstress conditions [53].

### 4.4. The Iron Exclusion Behavior Reveals an Important Tolerance Mechanism Which Would Be Helpful in the Rehabilitation of Iron Mining Areas

The iron exclusion ability of the *P. cinerascens* plants, notably the very high amounts of Fe retained in the belowground tissues instead of being transported to the shoots via the transpiration stream, may represent an important iron tolerance mechanism that, combined with high carbon assimilation, allows this species to grow and thrive in the *canga* physiognomies and also in the iron-rich mining substrates of Serra dos Carajás. Because of the equivalent iron concentration found in the leaves of *A. longispicus* in this study (although not analyzed in the root system), together with their common occurrence in *canga* physiognomies [20], we can expect that both species share the same strategy to deal with high iron availability. In fact, to reduce the metabolic risk associated with excess iron, species that adapt to environments naturally rich in metals can store large quantities of such elements by carrying the elements to the shoot organs (hyperaccumulators) or can prevent the elements from being absorbed in high quantities (excluders). A sophisticated combination of transport and accumulation in structures such as Fe-ferritin complexes is required in the former strategy to prevent the production of chemically reactive species (hydroxyl radicals) through the Fenton reaction [66], while in the latter mechanism is commonly found the formation of iron plaques around the roots as a consequence of the precipitation of Fe-oxyhydroxide (ferrihydrite), the restriction of iron uptake by apoplastic barriers, and Fe deposits in the root tissues (epidermal and cortical cells) in plants exposed to high iron availability [22,67]. Although in this study we did not evaluate the root anatomical structures, these adaptations are frequently observed in grasses, including the *Paspalum* genus. This tolerance mechanism is behind the spontaneous growth of the perennial grasses *Paspalum urvillei* Steudel and *Setaria parviflora*, found at the borders of the decantation ponds of the iron pelletizing industry [68] and the high iron tolerance of *Paspalum densum* (Poir.) and *Echinochloa crus-galli* (L.) P. Beauv. [67]. Therefore, this iron exclusion ability can also be an adaptive advantage shared by *P. cinerascens* and *A. longispicus* and may be important for rehabilitating metal-rich environments such as mining areas impacted by iron ore exploitation.

## 5. Conclusions

Both studied native grass species are able to grow and propagate in mining waste substrates and can contribute to the rehabilitation processes of degraded areas resulting from iron ore extraction. Nitrogen fertilization enhances the tillering of both species, which provides additional protection against soil erosion and ensures the propagation and survival of the species. While information regarding seed production and quality is still absent, vegetative propagation improvements can be an alternative way to produce large plant numbers of both species in nursery conditions.

Plants of the species *P. cinerascens* also benefited from N availability, which promptly improved their growth. With notable gains from the lower N levels and reaching an optimum growth in a mining waste substrate of approximately 80 mg of N dm^−3^, *P. cinerascens* plants were able to improve their pigment contents, carbon assimilation rates, and biomasses. A series of adaptations allowed these plants to achieve equivalent carbon assimilation rates, stomatal conductance, and water use efficiencies as those of field-growing plants in *canga* grasslands during the wet season. These adaptations, including stomatal morphology and frequency and the iron excluder strategy (trapping the iron excess in the roots), led *P. cinerascens* plants to efficiently use nutrients and save water, thereby, showing high potential to protect the soil throughout the seasons. Thus, in substrates depleted of organic matter and soil structure, an input of N as fertilizer can be of paramount importance to start rehabilitation processes by improving the growth of native species adapted to severe local conditions. These plants are able to assimilate and deposit high carbon quantities in the soil, and thus are promising candidates for restoring nutrient cycling and accelerating the return of other species and ecosystem services.

## Data Availability

The data presented in this study are available in the article and in the Supplementary Materials.

## Supplementary Materials

The following are available online at https://www.mdpi.com/article/10.3390/plants10050849/s1, Table S1: Chemical and physical properties of the red mining waste substrate collected from a representative location of the S11D Eliezer Batista Complex, Canaã dos Carajás, Pará/Brazil, Table S2: The regression parameters of 37 variables measured in the plants of *Paspalum cinerascens* cultivated in mining waste substrate in response to nitrogen addition, Table S3: The regression parameters of 26 variables measured in the plants of *Axonopus longispicus* cultivated in mining waste substrate in response to nitrogen addition, Figure S1: Mean air temperature and vapor pressure deficit inside the greenhouse during the 110 days of plants growing, Figure S2: Nitrogen growth response of two native grass species from *canga* in Serra dos Carajás (eastern Amazon) grown in mining waste substrate. The shoot and root plant dry mass of *Paspalum cinerascens* (blue) and *Axonopus longispicus* (red) are shown, Figure S3: Boxplots of the carbon assimilation rates of two native grass species (*Paspalum cinerascens* (blue) and *Axonopus longispicus* (red)) from *canga* in Serra dos Carajás (eastern Amazon) growing in the mining waste substrates without nitrogen addition (control treatment), Figure S4: Effects of nitrogen increment to mining waste substrate in the pigment contents of two native grass species from *canga* of Carajás (eastern Amazon). Chlorophyll a (A), chlorophyll b (B), the ratio between chlorophyl a/b (C), and carotenoids (D) from fresh leaf tissue of *Paspalum cinerascens* (circles, blue) and *Axonopus longispicus* (triangles, red). Lines correspond to best fitted model and shaded area to its confidence intervals, Figure S5: Plants of *Paspalum cinerascens* cultivated in a nutrient-rich substrate (a mixture of loam soil, organic matter, sand, and fertilizer) under greenhouse conditions. Images captured from (A) plants three-month-old, and (B) five-month-old.
