# Supplementary material for: Native Amazonian Canga Grasses Show Distinct Nitrogen Growth Responses in Iron Mining Substrates
Source: Plants (Basel). 2021 Apr 22;10(5):849. doi: 10.3390/plants10050849 (PMC8146357; doi:10.3390/plants10050849)
Supplement: Supplementary file 1 [file plants-10-00849-s001.zip › plants-1182422-supplementary.pdf]

**Manuscript title:** Native Amazonian *canga* grasses show distinct nitrogen growth responses in iron mining substrates

**Authors:** Cecilio F. Caldeira, Madson O. Lima, Silvio J. Ramos, Markus Gastauer

**Author affiliations:** Instituto Tecnológico Vale, Rua Boaventura da Silva 955, CEP 66055-090, Belém, Pará, Brazil

***Corresponding author:** cecilio.caldeira@itv.org, Instituto Tecnológico Vale. Boaventura da Silva, 955. Belém/PA, Brazil.

**TABLES**

**Table S1.** Chemical and physical properties of the red mining waste substrate collected from a representative location of the S11D Eliezer Batista Complex, Canaã dos Carajás, Pará/Brazil.

| pH | OC | N | P | K | S | B | Zn | Fe | Mn | Cu | Ca | Mg | Al | Sand | Clay | Silt |
|---|---|---|---|---|---|---|---|---|---|---|---|---|---|---|---|---|
| | % | | mg dm$^{-3}$ | | | | | | | | cmolc dm$^{-3}$ | | | % | | |
| 5.1 | 0.4 | <0.1 | 14.8 | 8.1 | 92.5 | 0.2 | 1.7 | 76.2 | 5.5 | 0.4 | 0.20 | 0.10 | <0.1 | 39.3 | 16.7 | 44.0 |

**TABLES**

**Table S2.** The regression parameters of 37 variables measured in the plants of *Paspalum cinerascens* cultivated in mining waste substrate in response to nitrogen addition.

| Species | Variable | Intercept | x | $x^{0.5}$ | $r^2$ | p.value |
|---|---|---|---|---|---|---|
| *P. cinerascens* | Tillering rate (tiller $plant^{-1}$) | 3.8410 | -0.0375 | 1.2333 | 0.6601 | 6.1E-08 |
| | Plant dry mass (g) | 7.2975 | -0.0855 | 2.1523 | 0.7829 | 9.0E-11 |
| | Root: shoot ratio (g $g^{-1}$) | 1.0289 | 0.0041 | -0.0859 | 0.3563 | 6.4E-04 |
| | A - carbon assimilation (μmolCO2 $m^{-2}$ $s^{-1}$) | 10.5460 | -0.0737 | 1.8827 | 0.7246 | 2.2E-16 |
| | gs - stomatal conductance (mol$H_2O$ $m^{-2}$ $s^{-1}$) | 0.1232 | -0.0007 | 0.0171 | 0.7475 | 2.2E-16 |
| | WUE - water use efficiency (μmol$CO_2$ mmol$H_2O^{-1}$) | 4.1605 | -0.0101 | 0.2291 | 0.3824 | 2.2E-16 |
| | Chlorophyl a (mg $g^{-1}$ FW) | 4.0679 | 0.0177 | - | 0.5017 | 6.5E-05 |
| | Chlorophyl b (mg $g^{-1}$ FW) | 2.5033 | 0.0135 | - | 0.6777 | 4.7E-07 |
| | Carotenoids (mg $g^{-1}$ FW) | 1.5037 | 0.0048 | - | 0.4446 | 2.2E-04 |
| | N shoot (g $kg^{-1}$) | 4.7112 | -0.0334 | 0.5969 | 0.4166 | 2.2E-16 |
| | N root (g $kg^{-1}$) | 4.4092 | -0.0265 | 0.4059 | 0.2923 | 6.3E-04 |
| | P shoot (g $kg^{-1}$) | 0.6358 | -0.0001 | - | -0.0054 | 6.2E-01 |
| | P root (g $kg^{-1}$) | 0.4890 | 0.0003 | - | 0.0088 | 2.5E-01 |
| | K shoot (g $kg^{-1}$) | 10.0548 | -0.0195 | - | 0.5369 | 2.2E-16 |
| | K root (g $kg^{-1}$) | 8.0553 | -0.0138 | - | 0.2257 | 1.1E-03 |
| | Ca shoot (g $kg^{-1}$) | 2.2360 | -0.0022 | - | 0.0974 | 1.1E-04 |
| | Ca root (g $kg^{-1}$) | 1.5192 | -0.0069 | 0.0857 | 0.1607 | 1.5E-02 |
| | Mg shoot (g $kg^{-1}$) | 1.0245 | -0.0083 | 0.1723 | 0.2843 | 4.9E-11 |
| | Mg root (g $kg^{-1}$) | 1.2799 | -0.0094 | 0.1562 | 0.2561 | 1.6E-03 |
| | S shoot (g $kg^{-1}$) | 3.5299 | 0.0096 | -0.2952 | 0.7768 | 2.2E-16 |
| | S root (g $kg^{-1}$) | 2.1386 | 0.0040 | - | 0.3406 | 4.6E-05 |
| | B shoot (mg $kg^{-1}$) | 11.0089 | 0.0082 | -0.3289 | 0.1548 | 4.0E-06 |
| | B root (mg $kg^{-1}$) | 8.1800 | 0.0464 | 0.9599 | 0.5088 | 7.3E-07 |
| | Cu shoot (mg $kg^{-1}$) | 7.0579 | 0.0977 | -0.7792 | 0.5511 | 2.2E-16 |
| | Cu root (mg $kg^{-1}$) | 9.3160 | 0.0274 | - | 0.1838 | 3.4E-03 |
| | Fe shoot (mg $kg^{-1}$) | 0.2550 | 0.0001 | -0.0090 | 0.1550 | 3.9E-06 |
| | Fe root (mg $kg^{-1}$) | 18.1 | 0.1024 | -1.4347 | 0.3303 | 2.3E-04 |
| | Mn shoot (mg $kg^{-1}$) | 0.1595 | 0.0002 | -0.0059 | 0.1555 | 3.8E-06 |
| | Mn root (mg $kg^{-1}$) | 0.0947 | 0.0006 | -0.0087 | 0.1696 | 1.2E-02 |
| | Zn shoot (mg $kg^{-1}$) | 29.0025 | -0.0869 | 0.7495 | 0.0983 | 3.2E-04 |
| | Zn root (mg $kg^{-1}$) | 22.8319 | -0.1196 | 1.8137 | 0.1080 | 4.6E-02 |
| | Stomatal frequence - adaxial | 10.0924 | 0.0088 | - | -0.0407 | 6.5E-01 |
| | Stomatal frequence - abaxial | 106.2524 | -0.2065 | - | 0.3673 | 1.7E-03 |
| | Stomatal length - adaxial | 29.8621 | 0.0271 | - | 0.3787 | 0.0014 |
| | Stomatal width - adaxial | 23.2206 | 0.0231 | - | 0.5230 | 8.6E-05 |
| | Stomatal length - abaxial | 23.6545 | 0.0495 | - | 0.4457 | 4.1E-04 |
| | Stomatal width - abaxial | 16.4847 | 0.0116 | - | 0.2854 | 6.1E-03 |

**TABLES**

**Table S3.** The regression parameters of 26 variables measured in the plants of *Axonopus longispicus* cultivated in mining waste substrate in response to nitrogen addition.

| Species | Variable | Intercept | x | $x^{0.5}$ | $r^2$ | p.value |
|---|---|---|---|---|---|---|
| | Tillering rate (tiller $plant^{-1}$) | 5.4200 | 0.0399 | - | 0.3025 | 6.5E-04 |
| | Plant dry mass (g) | 2.4204 | 0.0013 | - | 0.0239 | 6.0E-01 |
| | Root: shoot ratio (g $g^{-1}$) | 0.2663 | -0.0005 | - | -0.0327 | 8.9E-01 |
| | A - carbon assimilation (µmol$CO_2$ $m^{-2}$ $s^{-1}$) | 9.9732 | 0.0055 | - | 0.0174 | 4.3E-02 |
| | gs - stomatal conductance (mol$H_2O$ $m^{-2}$ $s^{-1}$) | 0.1009 | 0.0001 | - | 0.0604 | 5.1E-04 |
| | WUE - water use efficiency (µmol$CO_2$ mmol$H_2O^{-1}$) | 4.7480 | -0.0013 | - | 0.0017 | 2.6E-01 |
| | Chlorophyl a (mg $g^{-1}$ FW) | 2.3185 | 0.0052 | - | -0.0074 | 3.7E-01 |
| | Chlorophyl b (mg $g^{-1}$ FW) | 1.8453 | 0.0023 | - | -0.0334 | 5.8E-01 |
| | Carotenoids (mg $g^{-1}$ FW) | 0.7991 | 0.0051 | - | 0.1264 | 5.8E-02 |
| | N shoot (g $kg^{-1}$) | 8.9435 | 0.0177 | - | 0.2746 | 2.2E-11 |
| | P shoot (g $kg^{-1}$) | 0.4882 | 0.0014 | - | 0.1666 | 3.6E-07 |
| | K shoot (g $kg^{-1}$) | 8.4459 | 0.0141 | - | 0.1564 | 8.6E-07 |
| *A. longispicus* | Ca shoot (g $kg^{-1}$) | 1.8117 | 0.0021 | - | 0.2557 | 1.3E-10 |
| | Mg shoot (g $kg^{-1}$) | 2.0938 | 0.0015 | - | 0.0822 | 3.7E-04 |
| | S shoot (g $kg^{-1}$) | 2.7010 | 0.0051 | - | 0.1509 | 1.4E-09 |
| | B shoot (mg $kg^{-1}$) | 8.8717 | 0.0095 | - | 0.0817 | 3.8E-03 |
| | Cu shoot (mg $kg^{-1}$) | 10.0545 | 0.1828 | -1.6700 | 0.7677 | 2.2E-16 |
| | Fe shoot (mg $kg^{-1}$) | 0.4973 | -0.0008 | - | 0.0641 | 1.5E-03 |
| | Mn shoot (mg $kg^{-1}$) | 0.2946 | -0.0022 | 0.0376 | 0.3139 | 2.8E-12 |
| | Zn shoot (mg $kg^{-1}$) | 29.3572 | -0.0722 | 1.4001 | 0.2658 | 2.8E-10 |
| | Stomatal frequence - adaxial | 88.2948 | 0.0179 | - | -0.0505 | 7.7E-01 |
| | Stomatal frequence - abaxial | 91.9995 | -0.1171 | - | 0.4954 | 3.2E-04 |
| | Stomatal length - adaxial | 32.059 | -0.007 | - | 0.022 | 2.5E-01 |
| | Stomatal width - adaxial | 18.121 | -0.004 | - | 0.016 | 2.7E-01 |
| | Stomatal length - abaxial | 34.043 | 0.001 | - | -0.053 | 8.5E-01 |
| | Stomatal width - abaxial | 19.250 | 0.000 | - | -0.055 | 9.4E-01 |

**FIGURES**

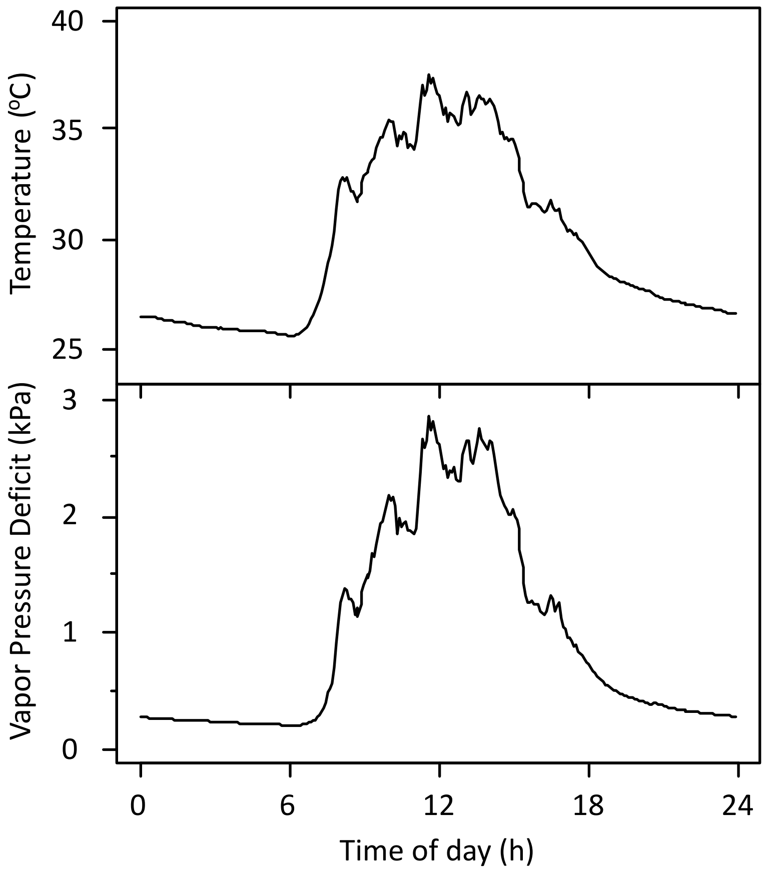


**Figure S1.** Mean air temperature and vapor pressure deficit inside the greenhouse during the 110 days of plants growing.

**FIGURES**

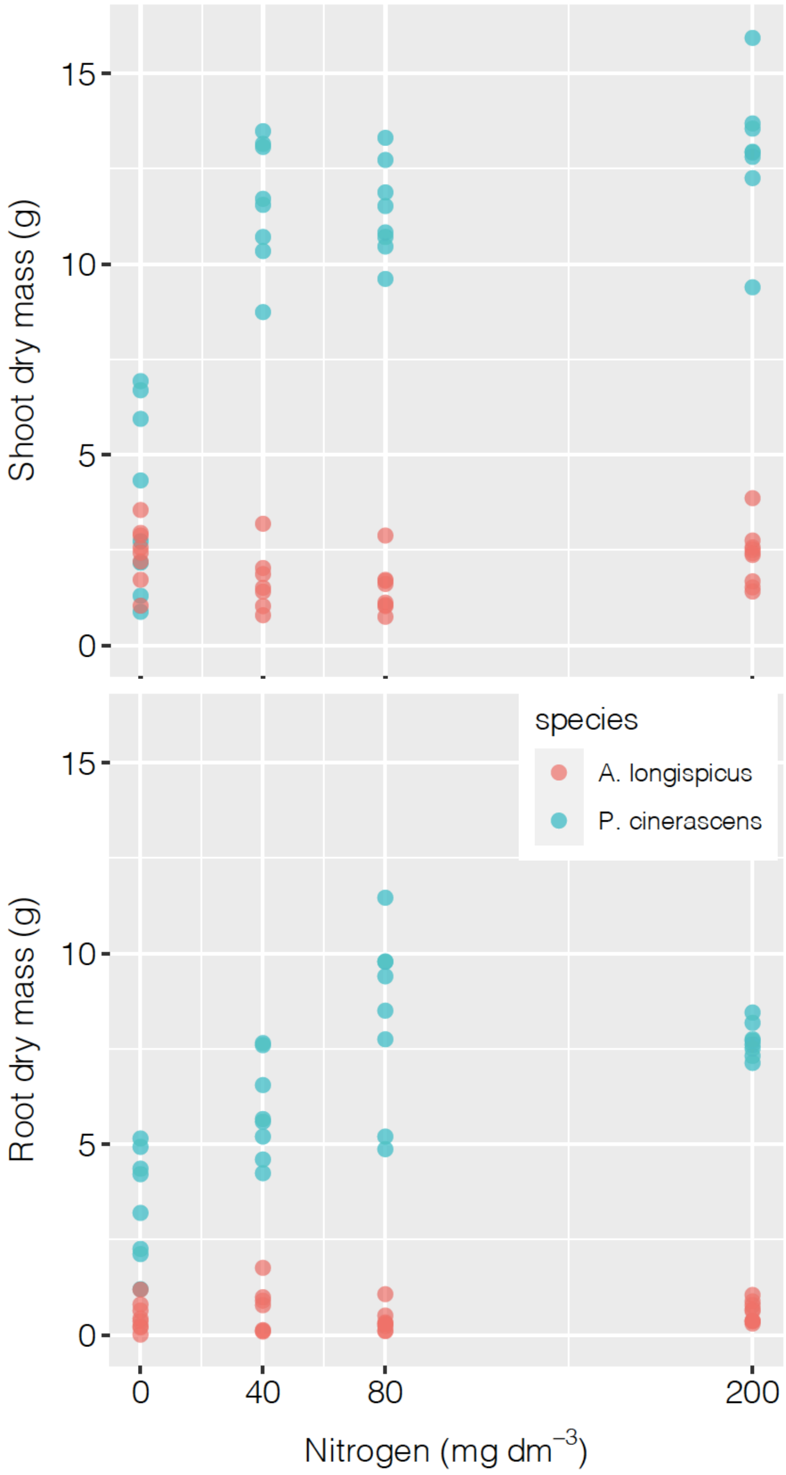


**Figure S2.** Nitrogen growth response of two native grass species from *canga* in Serra dos Carajás (eastern Amazon) grown in mining waste substrate. The shoot and root plant dry mass of *Paspalum cinerascens* (blue) and *Axonopus longispicus* (red) are shown.

**FIGURES**

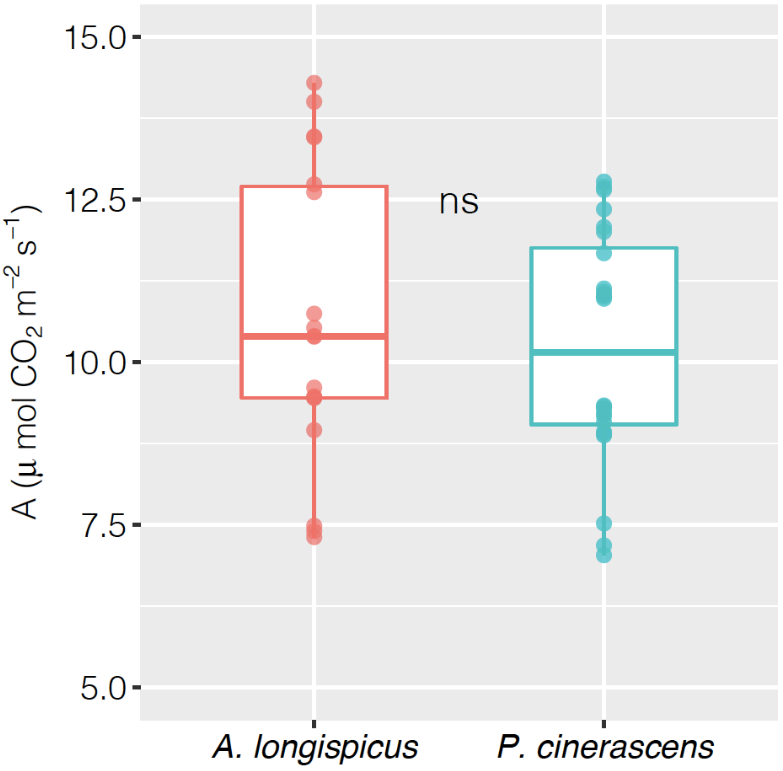


**Figure S3.** Boxplots of the carbon assimilation rates of two native grass species (*Paspalum cinerascens* (blue) and *Axonopus longispicus* (red)) from *canga* in Serra dos Carajás (eastern Amazon) growing in the mining waste substrates without nitrogen addition (control treatment).

**FIGURES**

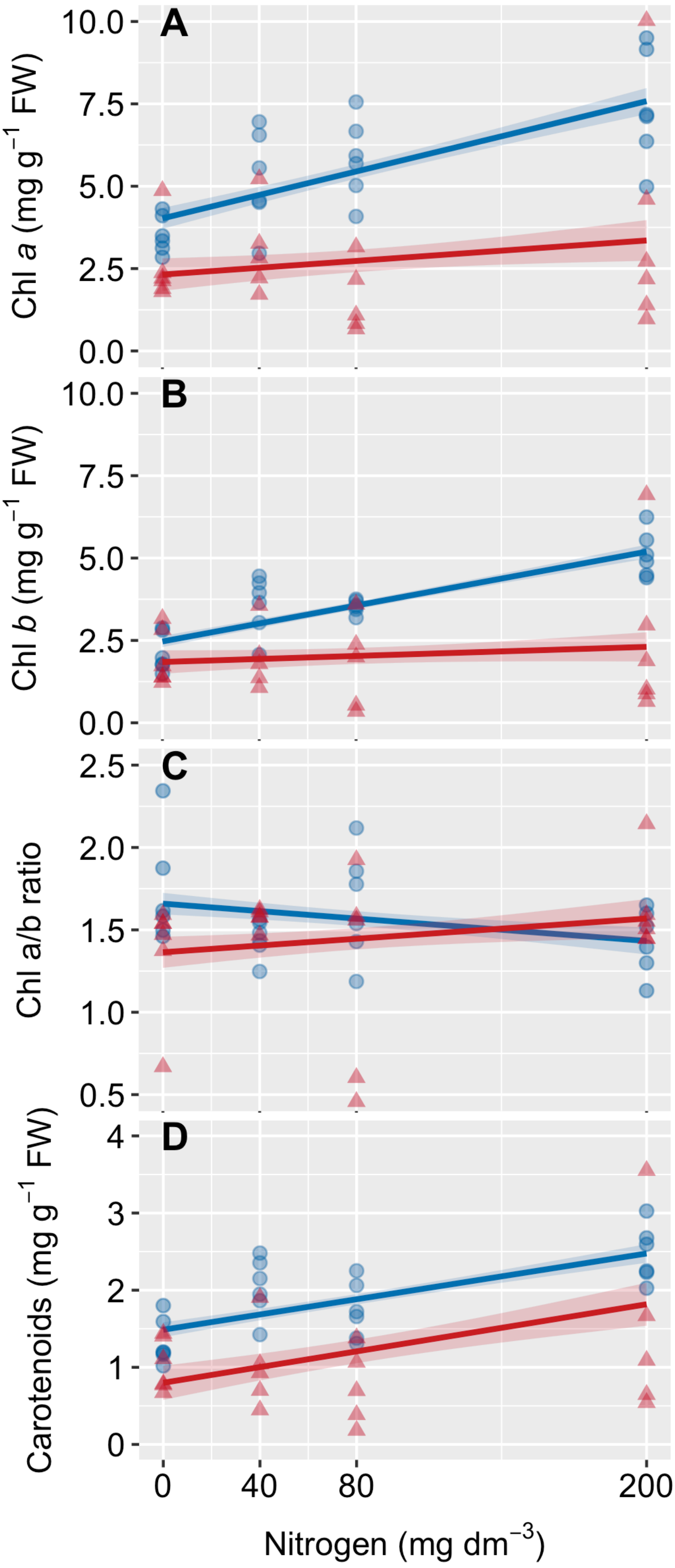


**Figure S4.** Effects of nitrogen increment to mining waste substrate in the pigment contents of two native grass species from canga of Carajás (eastern Amazon). Chlorophyll *a* (A), chlorophyll *b* (B), the ratio between chlorophyl *a/b* (C), and carotenoids (D) from fresh leaf tissue of *Paspalum cinerascens* (circles, blue) and *Axonopus longispicus* (triangles, red). Lines correspond to best fitted model and shaded area to its confidence intervals. Regression parameters are shown in the Supplemental Tables S2 and S3.

**FIGURES**

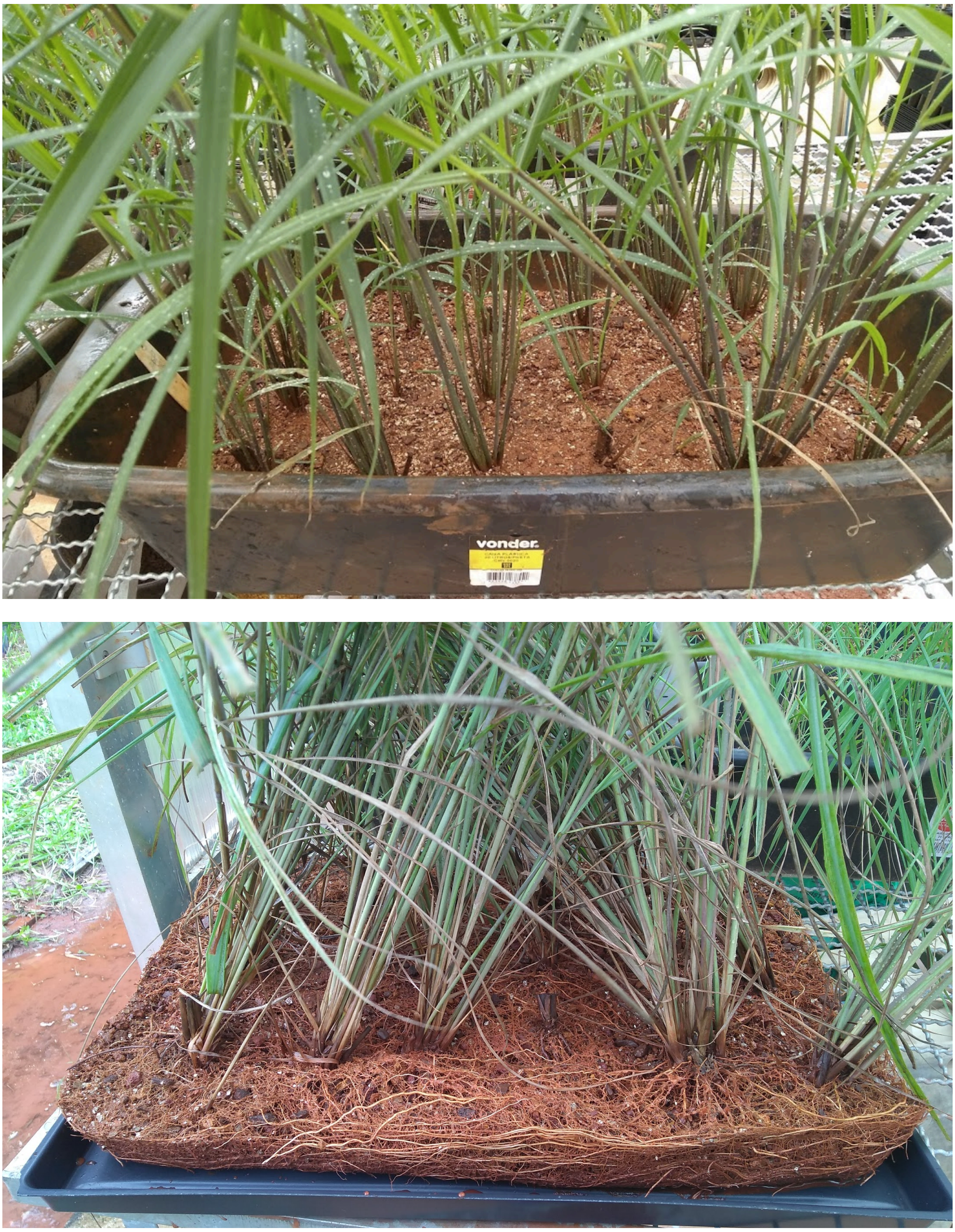


**Figure S5.** Plants of *Paspalum cinerascens* cultivated in a nutrient-rich substrate (a mixture of loam soil, organic matter, sand and fertilizer) under greenhouse conditions. Images captured from (A) plants three month-old, and (B) five month-old.
